# Prognostic benefit of immunosuppressive agents in primary Sjögren’s syndrome patients with hypergammaglobulinemia

**DOI:** 10.3389/fimmu.2026.1772604

**Published:** 2026-03-12

**Authors:** Liupan Zhang, Yiwen Zou, Dongyi Chen, Xin Zhang, Zhuoyang Jia, Qingfeng Zhang, Fan Wang, Xuebing Feng

**Affiliations:** 1 Department of Rheumatology and Immunology, Nanjing Drum Tower Hospital, Affiliated Hospital of Medical School, Nanjing University, Nanjing, Jiangsu, China; 2 Department of Critical Care Medicine, Nanjing Drum Tower Hospital, Affiliated Hospital of Medical School, Nanjing University, Nanjing, China

**Keywords:** adverse outcome, hypergammaglobulinemia, immunosuppressive agents, prognosis, Sjögren’s syndrome

## Abstract

**Objective:**

To elucidate the prognosis of patients with primary Sjögren’s syndrome (pSS) accompanied by hypergammaglobulinemia (HG), with a particular focus on the impact of various treatments.

**Methods:**

Patients were divided into the HG group and the non-HG group, based on the presence or absence of HG [serum immunoglobulin G (IgG) level above 16 g/L]. The demographics, clinical manifestations, laboratory findings, medications and outcomes were compared between the two groups. The adverse outcome was defined as death or the increase of Sjögren’s Syndrome Disease Damage Index (SSDDI) score.

**Results:**

366 patients were included, with 225 (61.5%) in the non-HG group and 141 (38.5%) in the HG group. Compared with the non-HG group, the HG group had a greater proportion of females, higher disease activity, higher frequencies of organ involvements (excluding interstitial lung disease), and higher rates of autoantibody positivity, hypocomplementemia, and elevated erythrocyte sedimentation rate. After a follow-up of 36 (28-45) months, adverse outcomes were observed in 14 of 141 (9.9%) patients in the HG group and 23 of 225 (10.2%) in the non-HG group. In the Kaplan-Meier curve analysis, the risk of adverse outcomes did not differ between the two groups either unadjusted or corrected for conditions such as disease activity. However, compared with non-HG group, the HG group had a lower risk of poor prognosis when using immunosuppressive agents (ISA), and a trend toward a higher risk of poor prognosis when not using (adjusted P for interaction = 0.01).

**Conclusion:**

Although the prognosis of pSS patients with HG was comparable to that of non-HG patients in this study, the application of ISA may help to improve the outcome of such patients.

## 1 Introduction

Primary Sjögren’s syndrome (pSS) is a slowly progressive autoimmune disorder characterized by lymphocyte infiltration of the salivary and lacrimal glands with concomitant damage to glandular tissue (1). Patients with pSS typically demonstrate biological signs of B cell activation including hypergammaglobulinemia (HG), elevated free light chain levels and autoantibody positivity (2). HG is the most common serological manifestation of pSS, occurring in approximately 33.6%-75% of patients (3–7). Most cases are polyclonal, with a few presenting monoclonal.

HG is typically associated with higher incidences of parotidomegaly and systemic involvements in pSS patients (3–5), and may predict progression of the disease (8), which has prompted rheumatologists to favor more aggressive treatment. As a result, such patients tend to be treated more often with glucocorticoids (GC), hydroxychloroquine (HCQ), and/or immunosuppressive agents (ISA) (3, 4, 9, 10), even if there is no evidence yet that HG is associated with a poor prognosis. Until recently, HG was identified as a mortality risk factor for pSS patients in a Chinese Rheumatic Disease Data Center (CRDC) study, with 5-, 10-, and 15-year survival rates of 96.9%, 92.3%, and 87.9%, respectively (9). However, in that paper, HG was defined as any elevated serum levels of IgG, immunoglobulin A (IgA), or immunoglobulin M (IgM), whereas we have already known that elevated monoclonal immunoglobulins or cryoglobulinemia were associated with lymphoma and death (4, 7, 11).

In this study, we focused on pSS patients with and without HG and followed up their clinical outcomes for a median time of 36 months, so as to provide a reference for better treatment of these patients.

## 2 Materials and methods

### 2.1 Study population

Hospitalized patients with a first-ever diagnosis of pSS at Affiliated Drum Tower Hospital of Nanjing University Medical School from January 2019 and December 2021, aged between 18 and 80 years old, were enrolled in this study; only inpatients were included in this cohort. All patients fulfilled the 2002 American-European Consensus Group (AECG) or 2016 American College of Rheumatology/European League against Rheumatism (ACR/EULAR) classification criteria for SS (12, 13). Exclusion criteria were (1) lack of gamma globulin data, (2) overlapping with other connective tissue diseases, (3) monoclonal globulinemia, (4) concomitant infection, lymphoproliferative diseases, severe cardiovascular, cerebrovascular, hepatic, or renal diseases unrelated to pSS, and (5) lactation or pregnancy. The study was approved by the Ethics Committee of the Affiliated Drum Tower Hospital of Nanjing University Medical School (No. 2023-545), and written informed consent was waived as this was a retrospective observational study.

### 2.2 Data collection and definition

The demographics, clinical manifestations, laboratory findings and medications of patients at their initial diagnosis were collected by charts review, and divided into HG group and non-HG group based on whether or not the serum IgG level was greater than 16 g/L. Organ involvement was determined according to European League against Rheumatism Sjögren’s Syndrome Disease Activity Index (ESSDAI), and clinical ESSDAI (ClinESSDAI), which excluded the biological domain from ESSDAI, was employed to evaluate disease activity (14). To assess organ damage and long-term disease outcomes, the Sjogren’s syndrome disease damage index (SSDDI) (15, 16) was measured. The systemic medications prescribed at diagnosis, including GC, HCQ, and ISA, were documented, and the doses of GC were converted to equal amounts of prednisone. ISA encompassed methotrexate, leflunomide, cyclophosphamide, azathioprine, cyclosporine A, tacrolimus, and mycophenolate mofetil.

Hypocomplementemia was defined as complement C3 below 0.8 g/L and/or C4 below 0.2 g/L. Positive labial gland biopsy was defined as grade ≥ 3 in the Chisholm and Mason classification. High C-reactive protein (CRP) was defined as CRP levels exceeding 6 mg/L and high erythrocyte sedimentation rate (ESR) was defined as ESR greater than 20 mm/h. Anemia was defined as hemoglobin < 120 g/L, neutropenia as neutrophil count < 1.5×10^9^/L, thrombocytopenia as platelets < 150×10^9^/L, and lymphopenia as lymphocytes < 1.0×10^9^/L.

### 2.3 Outcomes

All patients were followed up in January 2024 via clinical visit to clarify their status. The adverse outcome was defined as death or the increase of SSDDI score. Increased SSDDI score was defined as an elevation of ≥1 point compared with the baseline. Follow-up time was the interval from the date of diagnosis to the date of last follow-up (January 2024) or the date of death. Cases lost to follow-up were considered censored data in survival analysis.

### 2.4 Statistical analysis

Continuous variables were presented as mean with standard deviation (SD) or median with interquartile range (IQR), and were analyzed by t-test or Wilcoxon-Mann Whitney test. Categorical variables were expressed as numbers with percentages and were compared with chi-square test or Fisher exact test. Factors with p value < 0.05 in the univariate analysis were included in the multivariable logistic regression analysis to investigate the independent associated factors for HG, and missing values were filled with normal ones. In the analysis of adverse outcome, the event-free probabilities were compared using Kaplan-Meier curves and log-rank tests adjusted with inverse probability weights (17). The Kaplan-Meier curve was adjusted for potential confounding variables included age, sex, and ClinESSDAI. Stratified analyses in the Cox proportional hazard regression model were based on the use/non-use of GC, HCQ, and ISA. Adjusted values in the stratified analyses included age, sex, ClinESSDAI and the use of GC, HCQ, and ISA. All analyses were conducted with SPSS version 22.0 and R version 4.3.2. A two-sided *P* value less than 0.05 was defined as statistically significant.

## 3 Results

### 3.1 Characteristics of the patients

**Figure 1:**
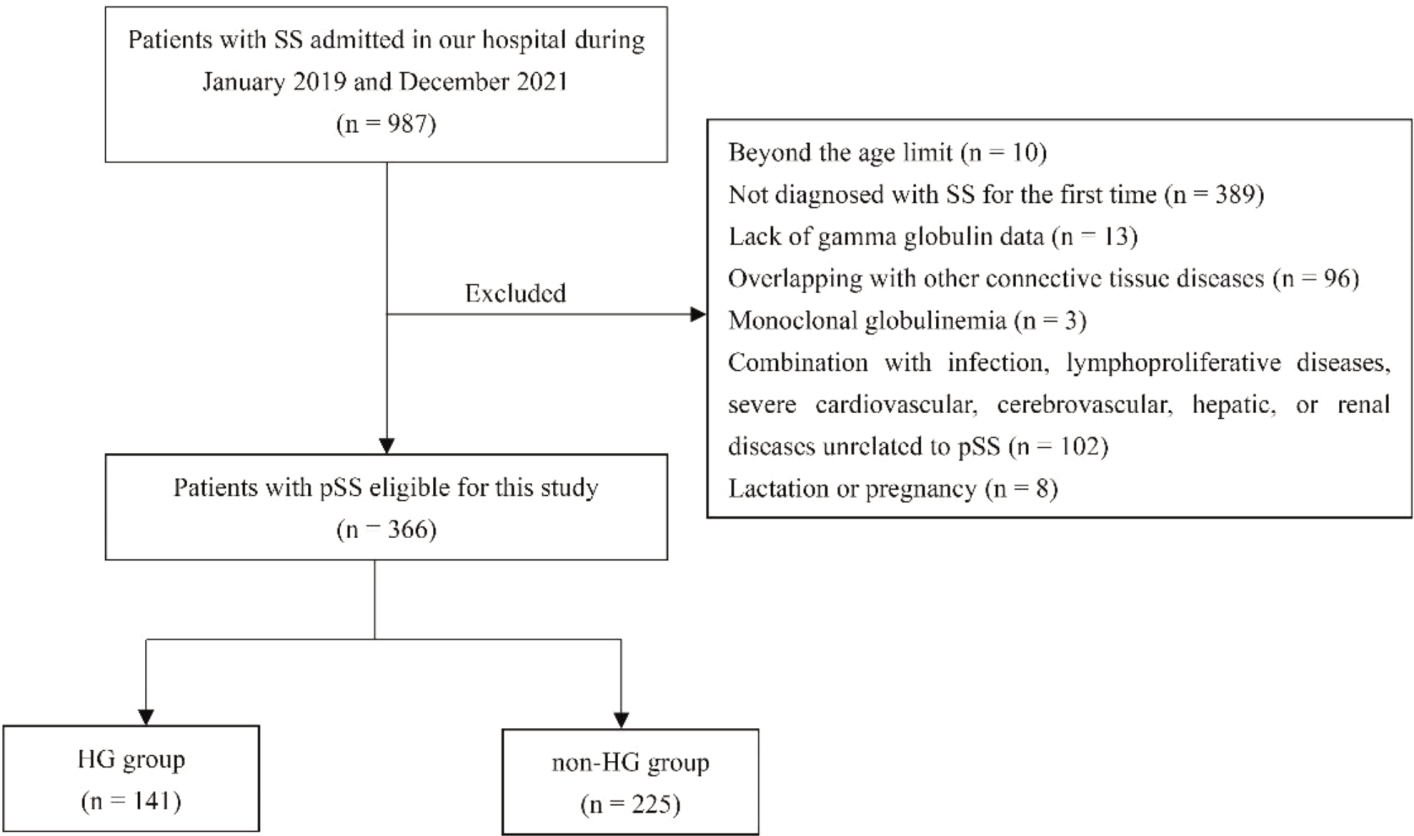
Follow-chart of patient selection.

As shown in **Figure 1**, 366 patients were included in the study, 225 (61.5%) in the non-HG group and 141 (38.5%) in the HG group. The demographic and clinical characteristics were listed in **Table 1**. The reasons for hospitalization were extra-glandular involvement (85.8%), sicca symptoms (9.6%) and other reasons (4.6%). Patients with HG presented a higher predominance of females (*P* = 0.01) and younger ages at diagnosis (*P* = 0.004) compared to those with non-HG. Some clinical manifestations were more frequent in HG group, such as Raynaud phenomenon (*P* < 0.001), lymphadenopathy (*P* < 0.001), and renal involvement (*P* < 0.001), except for a decreased trend in interstitial lung disease (ILD). Although ClinESSDAI scores were higher in the HG group (*P* < 0.001), SSDDI scores were comparable between the HG and non-HG groups (*P* > 0.05).

**Table 1:** Clinical characteristics and treatment of patients in the HG and non-HG group at baseline.

Variable	HG group (n = 141)	non-HG (n = 225)	*P*
Female, n (%)	133 (94.3)	192 (85.3)	0.01
Age, years, mean (SD)	52 (15)	57 (14)	0.004
Diabetes mellitus, n (%)	2 (1.4)	26 (11.6)	<0.001
Hypertension, n (%)	25 (17.7)	54 (24.0)	0.16
Dry mouth, n (%)	98 (69.5)	150 (66.7)	0.57
Dry eyes, n (%)	68 (48.2)	101 (44.9)	0.53
Constitutional symptoms, n (%)	48 (34.3)	60 (26.7)	0.12
Articular involvement, n (%)	40(28.4)	54 (24.0)	0.35
Glandular swelling, n (%)	8 (4.6)	4 (1.8)	0.07
Neuropathy, n (%)	6(4.3)	6 (2.7)	0.55
Cutaneous involvement, n (%)	21 (14.9)	27 (12.0)	0.43
Raynaud phenomenon, n (%)	21 (14.9)	8 (3.6)	<0.001
Lymphadenopathy, n (%)	81 (57.4)	61 (27.1)	<0.001
ILD, n (%)	38 (27.0)	82 (36.4)	0.06
Renal involvement, n (%)	54 (38.3)	37 (16.4)	<0.001
Muscular involvement, n (%)	8 (5.7)	6 (2.7)	0.14
ClinESSDAI, median (IQR)	14 (11-18)	8 (5-14)	<0.001
SSDDI, median (IQR)	2 (0-2)	1 (0-2)	0.15
Medications			
GC, n (%)	109 (77.3)	158 (70.2)	0.14
Prednisone dose, mg/day, median (IQR)	44 (20-50)	30 (12-50)	0.15
HCQ, n (%)	89 (63.1)	110 (48.9)	0.01
ISA, n (%)	91 (64.5)	124 (55.1)	0.08

ClinESSDAI, Clinical European League against Rheumatism Sjögren’s Syndrome Disease Activity Index; GC, glucocorticoids; HCQ, hydroxychloroquine; HG, hypergammaglobulinemia; ILD, interstitial lung disease; ISA, immunosuppressive agents; non-HG, non- hypergammaglobulinemia; SSDDI, Sjögren’s Syndrome Disease Damage Index.

In terms of systemic medications (**Table 1**), patients in the HG group were more likely to be prescribed GC (*P* = 0.14) and ISA (*P* = 0.08) than those in the non-HG group, although this was not statistically significant. The initial daily dose of GC was slightly higher in the non-HG group compared to its counterpart [44 (20-50) mg vs 30 (12-50) mg, *P* = 0.15]. A greater proportion of patients in the HG group received HCQ therapy than that in the non-HG group (63.1% vs 48.9%, *P* = 0.01).

### 3.2 Serologic abnormalities were more common in the HG group

**Table 2** showed the laboratory results of the study subjects. Compared with the non-HG group, the HG group had higher rates of hypocomplementemia (*P* < 0.001), anemia (*P* < 0.001), lymphopenia (*P* = 0.01), and higher levels of CRP (*P* = 0.003) and ESR (*P* < 0.001). More patients with HG were positive for anti-nuclear antibodies (ANA), anti-Ro60, anti-SSB, anti-Ro52 antibodies, and RF (all *P* < 0.001). There were no significant differences between the two groups with respect to the positive rates of labial gland biopsy, platelets, alanine aminotransferase, and estimated glomerular filtration rate.

**Table 2:** Laboratory findings of patients in the HG and non-HG group at baseline.

Variable	HG group (n = 141)	non-HG (n = 225)	*P*
Hypocomplementemia (%)	90 (63.8)	94 (41.8)	<0.001
ANA, positive (%)	126/131 (96.2)	160/200 (80.0)	<0.001
Anti-Ro60 antibody, positive (%)	104/133 (78.2)	95/204 (46.6)	<0.001
Anti-SSB antibody, positive (%)	52/133 (39.1)	28/203 (13.8)	<0.001
Anti-Ro52 antibody, positive (%)	117/130 (90.0)	116/204 (56.9)	<0.001
RF, positive (%)	77/126 (61.1)	36/196 (18.4)	<0.001
Schirmer’s test, positive (%)	27/57 (47.4)	43/80 (53.8)	0.46
Labial gland biopsy, positive (%)	58/81 (71.6)	87/131 (66.4)	0.43
High CRP (%)	51 (36.2)	49 (21.8)	0.003
High ESR (%)	123 (87.2)	98 (43.6)	<0.001
Anemia (%)	91 (64.5)	77 (34.2)	<0.001
Neutropenia (%)	15 (10.6)	16 (7.1)	0.25
Thrombocytopenia (%)	54 (38.3)	65 (28.9)	0.06
Lymphopenia (%)	53 (37.6)	54 (24.0)	0.01

ANA, anti-nuclear antibodies; CRP, C-reactive protein; ESR, erythrocyte sedimentation rate; HG, hypergammaglobulinemia; non-HG, non-hypergammaglobulinemia; RF, rheumatoidfactor.

### 3.3 HG was associated with disease activity in pSS patients

Multivariate logistic regression analysis (**Table 3**) were performed and showed female [odds ratio (OR) 3.03, 95% CI 1.01-9.11], Raynaud phenomenon (OR 4.97, 95% CI 1.37-17.98), lymphadenopathy (OR 2.09, 95%CI 1.07-4.08), ClinESSDAI (OR 1.11, 95% CI 1.04-1.17), hypocomplementemia (OR 2.07, 95% CI 1.10-3.91), anti-Ro60 antibody (OR 2.48, 95% CI 1.12-5.49), RF (OR 2.65, 95% CI 1.35-5.20), and high ESR (OR 4.46, 95% CI 2.11-9.45) as independent associated factors for HG (**Table 3**).

**Table 3:** Multivariable logistic regression analysis of associated factors for HG.

Variable	Multivariate model	
	OR (95% CI)	*P*
Sex (Female)	3.03 (1.01-9.11)	0.049
Age	0.99 (0.97-1.01)	0.22
Diabetes mellitus	0.37 (0.07-1.86)	0.23
Raynaud phenomenon	4.97 (1.37-17.98)	0.02
Lymphadenopathy	2.09 (1.07-4.08)	0.03
Renal involvement	1.46 (0.70-3.02)	0.31
ClinESSDAI	1.11 (1.04-1.17)	0.001
Hypocomplementemia	2.07 (1.10-3.91)	0.03
ANA, positivity	0.80 (0.32-2.02)	0.64
Anti-Ro60 antibody, positivity	2.48 (1.12-5.49)	0.03
Anti-SSB antibody, positivity	1.62 (0.72-3.62)	0.24
Anti-Ro52 antibody, positivity	2.30 (1.00-5.28)	0.05
RF, positivity	2.65 (1.35-5.20)	0.004
High CRP	1.00 (0.46-2.13)	0.99
High ESR	4.46 (2.11-9.45)	<0.001
Anemia	1.64 (0.87-3.07)	0.13
Lymphopenia	0.52 (0.26-1.05)	0.07

ANA, anti-nuclear antibodies; ClinESSDAI, Clinical European League against Rheumatism Sjögren’s Syndrome Disease Activity Index; CRP, C-reactive protein; ESR, erythrocyte sedimentation rate; HG, hypergammaglobulinemia; OR, odds ratio; RF, rheumatoidfactor.

### 3.4 Patients in the HG and non-HG groups had a similar prognosis

**Figure 2:**
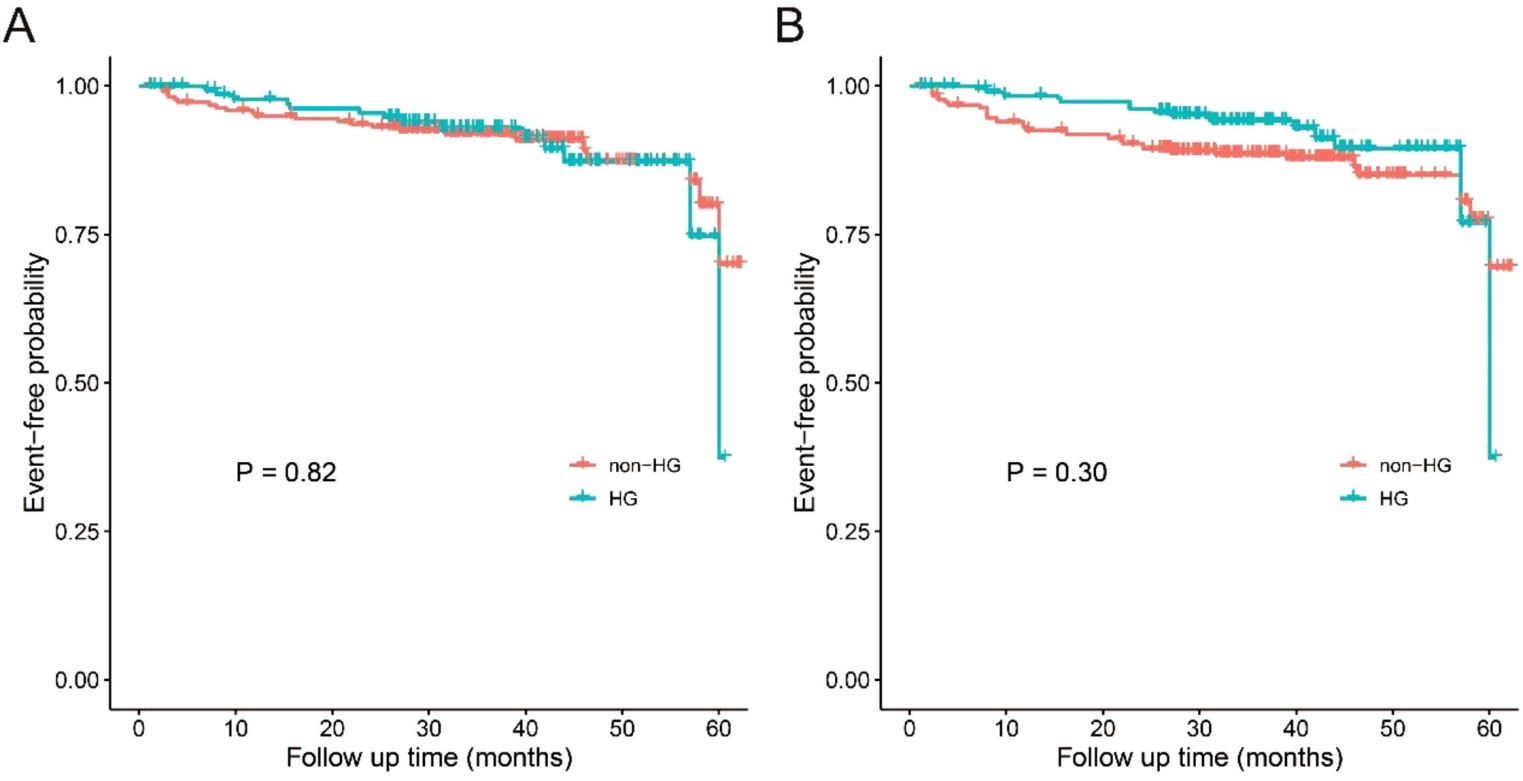
Kaplan-Meier curves for adverse outcomes in the HG and non-HG group. **(A)** unadjusted; **(B)** adjusted with inverse probability weights for age, sex, and ClinESSDAI. ClinESSDAI, Clinical European League against Rheumatism Sjögren’s Syndrome Disease Activity Index; HG, hypergammaglobulinemia; non-HG, non-hypergammaglobulinemia.

The overall follow-up time was 36 (28-45) months and there was no significant difference in follow-up time between the two groups (*P* = 0.76) (**Table 4**). 304 (83.1%) patients used these medications as prescribed. 62 (16.9%) patients had intermittent or discontinued treatment due to adverse drug reactions or poor therapeutic efficacy. During the follow-up, 25 deaths were recorded, with 8 in the HG group and 17 in the non-HG group. Of these, 13 were attributed to pulmonary infection (2 related to COVID-19), while the remaining patients died of malignancy (n = 4, one in the HG group related to cervical cancer and three in the non-HG group related to lymphoma or esophagus cancer), infections at other sites (n = 2), cardio-cerebral events (n = 4) and unknown causes (n = 2). Among the remaining 133 patients in the HG group, SSDDI scores were stable in 108 (81.2%), increased in 6 (4.5%), decreased in 7 (5.3%), and missing in 12 (9.0%). Among the remaining 208 patients in the non-HG group, SSDDI scores were stable in 181 (87.1%), increased in 6 (2.9%), decreased in 7 (3.3%), and missing in 14 (6.7%). Overall, adverse outcomes occurred in 14 of 141 (9.9%) and 23 of 225 (10.2%) patients in the HG and non-HG group, respectively. According to the Kaplan-Meier curve analysis (**Figure 2**), patients in the HG group presented comparable risk of adverse outcomes to those in the non-HG group, even after adjusting for age, sex, and ClinESSDAI (*P* > 0.05).

**Table 4:** Adverse outcomes in the HG and non-HG group.

Variable	HG group (n = 141)	non-HG group (n = 225)	*P*
Follow-up time, months, median (IQR)	35 (28-46)	37 (28-45)	0.76
Adverse outcomes	14	23	1.00
Death, n	8	17	
pulmonary infection, n	4	9	
Malignancy, n	1	3	
infections at other sites, n	0	2	
cardio-cerebral events, n	2	2	
unknown causes, n	1	1	
SSDDI increase, n	6	6	

HG, hypergammaglobulinemia; non-HG, non-hypergammaglobulinemia; SSDDI, Sjögren’s Syndrome Disease DamageIndex.

### 3.5 ISA was beneficial for pSS patients with HG

In order to explore the effect of various treatments on the outcomes of HG/non-HG patients, we conducted stratified analyses (**Table 5**). Based on the use/non-use of GC or HCQ, the risk of adverse outcomes in patients with HG was not significantly different from that in patients with non-HG (both *P* > 0.05). However, in the non-use of ISA subgroup, patients with HG presented a higher risk of adverse outcomes (*P* = 0.03); there was interaction between HG and the use/non-use of ISA (*P* for interaction = 0.02). After adjustment for age, sex, ClinESSDAI, the use of GC and HCQ, patients with HG had a higher risk of adverse outcomes in the non-use of ISA group (*P* = 0.07) and a lower risk in the use of ISA group (*P* = 0.03). The adjusted *P* for interaction between HG and the use/non-use of ISA was 0.01.

**Table 5:** Stratified analyses based on systemic medications in the adverse outcomes of HG/non-HG patients.

Variable	n (%)	HR (95% CI)	*P*	*P* for interaction	Adjusted HR (95% CI)	Adjusted *P*	Adjusted *P* for interaction
GC				0.45			0.55
No	99 (27.0)	0.62 (0.06-5.98)	0.68		0.31 (0.02-4.16)	0.37	
Yes	267 (72.0)	1.13 (0.55-2.31)	0.75		0.78 (0.34-1.81)	0.56	
HCQ				0.37			0.51
No	167 (45.6)	1.32 (0.49-3.55)	0.59		0.61 (0.18-2.05)	0.43	
Yes	199 (54.4)	0.85 (0.34-2.14)	0.74		0.70 (0.24-2.06)	0.52	
ISA				0.02			0.01
No	151 (41.3)	3.84 (1.11-13.24)	0.03		3.79 (0.90-15.88)	0.07	
Yes	215 (58.7)	0.56 (0.23-1.34)	0.19		0.30 (0.10-0.88)	0.03	

This table refers to both the HG group and the non-HG group. We performed Cox proportional hazards regression models. HR in the **Table 5** denotes the mortality risk of the HG group compared with the non−HG group. In the meantime, to explore the influence of treatment to the mortality risk, we performed stratified analyses based on the use or nonuse of different medications. Adjusted for age, sex, ClinESSDAI and the use of GC, HCQ, and ISA. The adjusted value would be excluded when it was a stratified value. ClinESSDAI, Clinical European League against Rheumatism Sjögren’s Syndrome Disease Activity Index; GC, glucocorticoids; HCQ, hydroxychloroquine; HG, hypergammaglobulinemia; ISA, immunosuppressive agents; non-HG, non-hypergammaglobulinemia.

## 4 Discussion

In this study, we compared the outcomes of pSS patients with polyclonal HG to those without HG and explored the effect of various treatment on their prognosis. The results showed that HG is prevalent in female pSS patients and is associated with disease activity and multiple serologic abnormalities. Although the prognosis of pSS patients with concomitant HG was quite similar to that of non-HG patients after following-up for about 36 months, ISA therapy may help to improve the prognosis of such patients.

B cell hyperactivation contributes to more infiltration of lymphocytes and increased deposition of immune complexes in inflamed tissues, which explains the greater incidence of lymphadenopathy, renal involvement and higher disease activity in the HG group (18, 19). Previous studies have employed ESSDAI to evaluate the long-term outcome of pSS patients (20, 21). However, although disease activity index is important to evaluate the current status of the inflammatory process, disease damage index seems to be more appropriate for assessing the chronic outcomes of recurrent disease flares and/or their therapies (15).

Despite the high prevalence of HG, its prognostic value has not been clearly elucidated. Two previous studies tried to investigate the correlation between HG and the long-term outcomes of extra-glandular involvement or damage and yielded conflicting results (5, 6). Neither of them showed the systemic involvement or damage at baseline and the change between the baseline and follow-up, which hindered the interpretation of patient outcomes. In previous mortality survival analyses, HG has mostly been excluded or left only in the ESSDAI rather than considered separately (22–24).

Our results showed that HG was not related to adverse outcomes, in line with a recent study (25). However, since patients with HG are usually prescribed aggressive treatment (3, 4, 9, 10), the prognosis of HG may be masked by treatment. In this study, we performed stratified analyses and found that ISA had a different impact on the prognosis of pSS patients with and without HG. Because this effect was different in the two subgroups, previous studies that typically considered the effects of drug treatment in the entire study population often struggled to uncover this phenomenon. ISA is primarily employed as GC-sparing agents (26). A recent study found that ISA/biotherapy could prevent the onset of systemic manifestations (27). ISA in this paper included a variety of drugs, yet it was difficult to analyze them separately due to the small sample sizes of individual drug applications. It is hypothesized that ISA may help alleviate chronic damage caused by the persistent inflammation in patients with HG.

Our data did not reveal a prognostic benefit of GC or HCQ application. The long-term use of HCQ is recommended in patients with systemic lupus erythematosus, due to the benefits of reducing mortality and using lower GC doses (28). However, evidence for the effect of HCQ in pSS is limited and controversial. A systemic review indicated that the effect of HCQ was not remarkable in improving HG and extra-glandular involvements (29). It has also been reported that HCQ was associated with better survival and seemed to protect against damage accrual in pSS patients, particularly at the pleuro-pulmonary domain (30, 31).

We acknowledge the limitations in this study. First, the small sample size, single-center, not long follow-up period, and retrospective nature of the study limited the interpretation of the results. Second, to facilitate systematic collection of complete clinical and follow-up data, and control for potential confounding factors from inconsistent clinical management in non-hospital settings, only hospitalized patients (no outpatients) were enrolled in this study. This inevitably results in a selection bias toward more severe patients. Therefore, our study can only provide some insights for inpatients. Third, unjustified treatment indication and treatment discontinuation also limit the interpretation of our findings. Besides, regardless of our best efforts, information on survival outcomes was still missing for 26 patients (7.0%). More importantly, the interpretation of results was based on correlation instead of causality. Thus, further studies with larger cohorts and longer follow-up are warranted.

## 5 Conclusion

Although the prognosis of pSS patients with HG was comparable to that of non-HG patients in this study, the application of ISA may help to improve the outcome of such patients. This finding requires confirmation in larger prospective clinical trials.

## Data Availability

The raw data supporting the conclusions of this article will be made available by the authors, without undue reservation.

## References

[1] Moriyama M Hayashida JN Toyoshima T Ohyama Y Shinozaki S Tanaka A . Cytokine/chemokine profiles contribute to understanding the pathogenesis and diagnosis of primary Sjogren’s syndrome. Clin Exp Immunol. (2012) 169:17–26. doi: 10.1111/j.1365-2249.2012.04587.x, PMID: 22670774 PMC3390469

[2] Nocturne G Mariette X . B cells in the pathogenesis of primary Sjogren syndrome. Nat Rev Rheumatol. (2018) 14:133–45. doi: 10.1038/nrrheum.2018.1, PMID: 29416129

[3] Koh JH Park Y Lee J Park SH Kwok SK . Hypergammaglobulinaemia predicts glandular and extra-glandular damage in primary Sjogren’s syndrome: results from the KISS cohort study. Clin Exp Rheumatol. (2021) 39 Suppl 133:114–22. doi: 10.55563/clinexprheumatol/volsh1, PMID: 34796856

[4] Martel C Gondran G Launay D Lalloue F Palat S Lambert M . Active immunological profile is associated with systemic Sjogren’s syndrome. J Clin Immunol. (2011) 31:840–7. doi: 10.1007/s10875-011-9553-3, PMID: 21744183

[5] Tsukamoto M Suzuki K Takeuchi T . Ten-year observation of patients with primary Sjogren’s syndrome: Initial presenting characteristics and the associated outcomes. Int J Rheum Dis. (2019) 22:929–33. doi: 10.1111/1756-185X.13464, PMID: 30588773

[6] Ter Borg EJ Kelder JC . Is extra-glandular organ damage in primary Sjogren’s syndrome related to the presence of systemic auto-antibodies and/or hypergammaglobulinemia? A long-term cohort study with 110 patients from the Netherlands. Int J Rheum Dis. (2017) 20:875–81. doi: 10.1111/1756-185X.13070, PMID: 28447402

[7] Fauchais AL Martel C Gondran G Lambert M Launay D Jauberteau MO . Immunological profile in primary Sjogren syndrome: clinical significance, prognosis and long-term evolution to other auto-immune disease. Autoimmun Rev. (2010) 9:595–9. doi: 10.1016/j.autrev.2010.05.004, PMID: 20457283

[8] Shiboski CH Baer AN Shiboski SC Lam M Challacombe S Lanfranchi HE . Natural history and predictors of progression to Sjogren’s syndrome among participants of the Sjogren’s international collaborative clinical alliance registry. Arthritis Care Res (Hoboken). (2018) 70:284–94. doi: 10.1002/acr.23264, PMID: 28437595 PMC5654699

[9] Zhong H Wang Y Yang P Duan X Wang Y Xu J . Hyperglobulinemia predicts increased risk of mortality in primary Sjogren’s syndrome: based on a Chinese multicentre registry. Mod Rheumatol. (2023) 34:137–43. doi: 10.1093/mr/road010, PMID: 36688590

[10] Baldini C Pepe P Quartuccio L Priori R Bartoloni E Alunno A . Primary Sjogren’s syndrome as a multi-organ disease: impact of the serological profile on the clinical presentation of the disease in a large cohort of Italian patients. Rheumatol (Oxford). (2014) 53:839–44. doi: 10.1093/rheumatology/ket427, PMID: 24369420

[11] Voulgarelis M Skopouli FN . Clinical, immunologic, and molecular factors predicting lymphoma development in Sjogren’s syndrome patients. Clin Rev Allergy Immunol. (2007) 32:265–74. doi: 10.1007/s12016-007-8001-x, PMID: 17992593

[12] Vitali C Bombardieri S Jonsson R Moutsopoulos HM Alexander EL Carsons SE . Classification criteria for Sjogren’s syndrome: a revised version of the European criteria proposed by the American-European Consensus Group. Ann Rheum Dis. (2002) 61:554–8. doi: 10.1136/ard.61.6.554, PMID: 12006334 PMC1754137

[13] Shiboski CH Shiboski SC Seror R Criswell LA Labetoulle M Lietman TM . 2016 American College of Rheumatology/European League Against Rheumatism classification criteria for primary Sjogren’s syndrome: A consensus and data-driven methodology involving three international patient cohorts. Ann Rheum Dis. (2017) 76:9–16. doi: 10.1136/annrheumdis-2016-210571, PMID: 27789466

[14] Seror R Meiners P Baron G Bootsma H Bowman SJ Vitali C . Development of the ClinESSDAI: a clinical score without biological domain. A tool Biol Stud Ann Rheum Dis. (2016) 75:1945–50. doi: 10.1136/annrheumdis-2015-208504, PMID: 27150113

[15] Seror R Bootsma H Bowman SJ Dorner T Gottenberg JE Mariette X . Outcome measures for primary Sjogren’s syndrome. J Autoimmun. (2012) 39:97–102. doi: 10.1016/j.jaut.2012.01.013, PMID: 22365784

[16] Vitali C Palombi G Baldini C Benucci M Bombardieri S Covelli M . Sjogren’s Syndrome Disease Damage Index and disease activity index: scoring systems for the assessment of disease damage and disease activity in Sjogren’s syndrome, derived from an analysis of a cohort of Italian patients. Arthritis Rheum. (2007) 56:2223–31. doi: 10.1002/art.22658, PMID: 17599741

[17] Cole SR Hernan MA . Adjusted survival curves with inverse probability weights. Comput Methods Programs BioMed. (2004) 75:45–9. doi: 10.1016/j.cmpb.2003.10.004, PMID: 15158046

[18] Francois H Mariette X . Renal involvement in primary Sjogren syndrome. Nat Rev Nephrol. (2016) 12:82–93. doi: 10.1038/nrneph.2015.174, PMID: 26568188

[19] Cornec D Devauchelle-Pensec V Tobon GJ Pers JO Jousse-Joulin S Saraux A . B cells in Sjogren’s syndrome: from pathophysiology to diagnosis and treatment. J Autoimmun. (2012) 39:161–7. doi: 10.1016/j.jaut.2012.05.014, PMID: 22749831

[20] Anquetil C Hachulla E Machuron F Mariette X Le Guern V Vittecoq O . Is early-onset primary Sjogren’s syndrome a worse prognosis form of the disease? Rheumatol (Oxford). (2019) 58:1163–7. doi: 10.1093/rheumatology/key392, PMID: 30561748

[21] Nguyen Y Nocturne G Henry J Ng WF Belkhir R Desmoulins F . Identification of distinct subgroups of Sjogren’s disease by cluster analysis based on clinical and biological manifestations: data from the cross-sectional Paris-Saclay and the prospective ASSESS cohorts. Lancet Rheumatol. (2024) 6:e216–25. doi: 10.1016/S2665-9913(23)00340-5, PMID: 38437852 PMC10949202

[22] Ioannidis JP Vassiliou VA Moutsopoulos HM . Long-term risk of mortality and lymphoproliferative disease and predictive classification of primary Sjogren’s syndrome. Arthritis Rheum. (2002) 46:741–7. doi: 10.1002/art.10221, PMID: 11920410

[23] Brito-Zeron P Flores-Chavez A Horvath IF Rasmussen A Li X Olsson P . Mortality risk factors in primary Sjogren syndrome: a real-world, retrospective, cohort study. EClinicalMedicine. (2023) 61:102062. doi: 10.1016/j.eclinm.2023.102062, PMID: 37457113 PMC10344811

[24] Brito-Zeron P Kostov B Solans R Fraile G Suarez-Cuervo C Casanovas A . Systemic activity and mortality in primary Sjogren syndrome: predicting survival using the EULAR-SS Disease Activity Index (ESSDAI) in 1045 patients. Ann Rheum Dis. (2016) 75:348–55. doi: 10.1136/annrheumdis-2014-206418, PMID: 25433020

[25] Yueting L Lin Q Jian X Xinwang D Yongfu W Weiguo X . Long-term survival analysis of patients with primary Sjogren’s syndrome in China: A multicenter retrospective cohort study. Int J Rheum Dis. (2024) 27:e15284. doi: 10.1111/1756-185X.15284, PMID: 39278720

[26] Ramos-Casals M Brito-Zeron P Bombardieri S Bootsma H De Vita S Dörner T . EULAR recommendations for the management of Sjogren’s syndrome with topical and systemic therapies. Ann Rheum Dis. (2020) 79:3–18. doi: 10.1136/annrheumdis-2019-216114, PMID: 31672775

[27] Belbezier A Nguyen TTT Arnaud M Ducotterd B Vangout M Deroux A . Treatment of non-systemic Sjogren’s syndrome: Potential prevention of systematization with immunosuppressant agent/biotherapy. J Transl Autoimmun. (2024) 8:100238. doi: 10.1016/j.jtauto.2024.100238, PMID: 38496268 PMC10940795

[28] Pawlak-Bus K Leszczynski P . Hydroxychloroquine as an important immunomodulator: a novel insight into an old drug. Pol Arch Intern Med. (2024) 134. doi: 10.20452/pamw.16656, PMID: 38166607

[29] Wang X Zhang T Guo Z Pu J Riaz F Feng R . The efficiency of hydroxychloroquine for the treatment of primary Sjogren’s syndrome: A systematic review and meta-analysis. Front Pharmacol. (2021) 12:693796. doi: 10.3389/fphar.2021.693796, PMID: 34588979 PMC8475756

[30] Gheitasi H Kostov B Solans R Fraile G Suarez-Cuervo C Casanovas A . How are we treating our systemic patients with primary Sjogren syndrome? Analysis of 1120 patients. Int Immunopharmacol. (2015) 27:194–9. doi: 10.1016/j.intimp.2015.03.027, PMID: 25899085

[31] Hernandez-Molina G Valim V Secco A Atisha-Fregoso Y Guerra E Adrover M . Do antimalarials protect against damage accrual in primary Sjogren’s syndrome? Results from a Latin-American retrospective cohort. Clin Exp Rheumatol. (2018) 36 Suppl 112:182–5. 29745873

